# Circulating testosterone and dehydroepiandrosterone are associated with individual motor unit features in untrained and highly active older men

**DOI:** 10.1007/s11357-021-00482-3

**Published:** 2021-12-03

**Authors:** Yuxiao Guo, Jessica Piasecki, Agnieszka Swiecicka, Alex Ireland, Bethan E. Phillips, Philip J. Atherton, Daniel Stashuk, Martin K. Rutter, Jamie S. McPhee, Mathew Piasecki

**Affiliations:** 1 MRC-Versus Arthritis Centre for Musculoskeletal Ageing Research and NIHR Nottingham BRC, School of Medicine, University of Nottingham, Nottingham, UK; 2 Musculoskeletal Physiology Research Group, Sport, Health and Performance Enhancement Research Centre, Nottingham Trent University, Nottingham, UK; 3 Division of Diabetes, Endocrinology and Gastroenterology, School of Medical Sciences, Faculty of Biology, Medicine and Health, University of Manchester, Manchester, UK; 4 Department of Basic and Clinical Sciences, University of Nicosia Medical School, Nicosia, Cyprus; 5 Department of Sport and Exercise Sciences, Musculoskeletal Science and Sports Medicine Research Centre, Manchester Metropolitan University, Manchester, UK; 6 Department of Systems Design Engineering, University of Waterloo, Waterloo, ON Canada; 7 Diabetes, Endocrinology and Metabolism Centre, Manchester University NHS Foundation Trust, Manchester Academic Health Science Centre, Manchester, UK

**Keywords:** Circulating sex hormones, Androgens, Electromyography, Muscle, Motor unit, Masters athletes

## Abstract

Long-term exercise training has been considered as an effective strategy to counteract age-related hormonal declines and minimise muscle atrophy. However, human data relating circulating hormone levels with motor nerve function are scant. The aims of the study were to explore associations between circulating sex hormone levels and motor unit (MU) characteristics in older men, including masters athletes competing in endurance and power events. Forty-three older men (mean ± *SD* age: 69.9 ± 4.6 years) were studied based on competitive status. The serum concentrations of dehydroepiandrosterone (DHEA), total testosterone (T) and estradiol were quantified using liquid chromatography mass spectrometry. Intramuscular electromyographic signals were recorded from vastus lateralis (VL) during 25% of maximum voluntary isometric contractions and processed to extract MU firing rate (FR), and motor unit potential (MUP) features. After adjusting for athletic status, MU FR was positively associated with DHEA levels (*p* = 0.019). Higher testosterone and estradiol were associated with lower MUP complexity; these relationships remained significant after adjusting for athletic status (*p* = 0.006 and *p* = 0.019, respectively). Circulating DHEA was positively associated with MU firing rate in these older men. Higher testosterone levels were associated with reduced MUP complexity, indicating reduced electrophysiological temporal dispersion, which is related to decreased differences in conduction times along axonal branches and/or MU fibres. Although evident in males only, this work highlights the potential of hormone administration as a therapeutic interventional strategy specifically targeting human motor units in older age.

**Supplementary Information:**

The online version contains supplementary material available at 10.1007/s11357-021-00482-3.

## Introduction

Ageing of the neuromuscular system is a complex process encompassing numerous pathophysiological conditions which is further compounded by sedentary behaviour [1]. The failure to maintain regular physical exercise while advancing in age not only induces weakness of the extremities [2] but also increases the probability of developing chronic disease [3] and associated co-morbidities [4]. As such, masters athletes provide a useful model to examine the effects of inherent ageing disassociated from negative factors such as physical inactivity [5]. Although some aspects of muscle strength may be maintained in masters athletes when compared to age-matched controls [6], this finding is equivocal [7] and progressive muscle atrophy demonstrates that lifelong exercise does not completely offset the muscle mass and strength decline caused by ageing [8]. A range of additional factors are involved, such as circulating sex hormones, which is further mediated by levels and/or types of physical activity [9]. Collectively, these factors also influence neural adaptations with age.

The final element of the peripheral motor nervous system related to muscle contraction is the motor unit (MU), consisting of an efferent motor neuron and the unique set of muscle fibres it innervates [10]. MUs undergo adaptive responses to external stimuli, most notably a decreased number with advancing age, leaving some fibres denervated [11, 12]. However, the surviving MUs have the ability to rescue recently denervated adjacent muscle fibres via collateral axonal sprouting and formation of new neuromuscular junctions (NMJ) [13]. By recording the electrical activity of muscles with intramuscular electrodes during voluntary contractions, a number of parameters relating to the structure and function of MUs can be investigated, including estimates of size, number and synchronicity of individual fibre activation [14].

Evidence for the preservation of MU number in human lifelong exercisers is ambiguous, with a study evidencing for [15] and others against [16–18] this notion. However, there is further evidence indicating a higher level of reinnervation ability in highly active older people. Compared with non-trained age-matched individuals, masters athletes exhibited larger motor unit potentials (MUPs), fewer denervated muscle fibres, and increased fibre type grouping [15, 19–21]. Thus, there are established benefits of exercise for the ageing neuromuscular system; yet, the interactions with circulating sex hormones in these highly active older individuals are unclear.

Testosterone (T) is the primary androgenic hormone and a precursor to estrogen synthesis [22], and has an anabolic impact on skeletal muscle [23]. Dehydroepiandrosterone (DHEA), the precursors of T and its 3-sulfooxy derivative (DHEAS), as well as the dihydrotestosterone (DHT) synthesised from T, have been reported to progressively decrease with ageing in men [24–28]. Estrogens are primarily produced by the ovaries in women but can also be synthesised in men through aromatization of T to estradiol (E2) in brain and adipose tissue [29], which also contributes to maintenance of muscle via estradiol receptors [30, 31].

To counteract age-related hormonal declines, exercise training [32–35] and exogenous hormone administration have been employed in several studies as an interventional strategy in older men and women [36–38]. Resistance exercise training acutely elevates T concentrations [39], and a recent study combining middle-aged (mean age 51 years) endurance and power athletes found them to have higher T than age-matched inactive subjects [40], suggesting athletic status directly influenced hormone levels in this age group. However, young individuals who regularly undergo endurance training have been reported to have a lower level of sex hormones compared to age-matched sedentary controls [41, 42]. In vitro models demonstrate that T treatment plays a neuroprotective role against the deprivation-mediated apoptosis of human neurons [43], and animal studies have shown that the atrophy of motoneuron dendrites could be attenuated or even reversed by T administration [44–46]. Both pre- and post-synaptic elements of the NMJ have been improved after T administration, independent of muscle fibre atrophy/hypertrophy, suggesting that it may largely contribute to the enhancement of NMJ transmission stability [47, 48]. Additionally, exogenous E2 administration has been reported to increase axonal regeneration [49–51].

We have previously highlighted the potential role of androgens in peripheral neuroplasticity via associations between circulating sex hormones and electrophysiological markers of MU function in frail elderly men [52], and separately, the effects of long-term athletic training on MU remodelling, specifically, the improved capacity to reinnervate denervated fibres in older age [53]. There is, however, limited data describing the influence of athletic status on relationship between hormones and MU function. The aims of the present study were therefore to investigate the effects of different lifelong exercise modalities on circulating sex hormone levels and neuromuscular properties, and to explore whether athletic status influences the associations between circulating sex hormone levels and MU characteristics of the vastus lateralis (VL) muscle in older men. We hypothesised that higher concentrations of circulating sex hormones would be observed in masters athletes and the athletic status would influence the associations between hormones and MU properties.

## Methods

### Ethical approval

This study was approved by Manchester Metropolitan University Research Ethics Committee and the National Research Ethics Service Committee Northwest (15/NW/4026) in accordance with the Declaration of Helsinki.

### Participants

A total of 43 males aged between 60 and 85 years were recruited between 2014 and 2017. This included 18 untrained controls (CON), 14 endurance masters athletes (EMA) and 11 power masters athletes (PMA). The controls, defined as recreationally active, did not take part in any form of regular and/or intensive exercise training and were recruited from the local communities. The athletes were recruited from running clubs and national masters athletic competitions, as well as through an advertisement in a national athletics magazine. At the time of testing, all masters athletes were regularly competing within their discipline and were completing more than 5 h of specified training per week. Power athletes were defined as those that were competing and training in running events less than 800 m along with throw and jump events. Endurance athletes were defined as those competing in running events greater than or equal to 800 m in distance. Mean age-graded performance (AGP) was determined by taking the athlete’s highest ranked performance within the last 2 years and expressing it as a percentage of the world record for that age and distance. The AGP was 79 ± 10% for EMA and 85 ± 10% for PMA, indicating a high level of performance relative to respective age group records. All masters athletes had been training and competing specifically within their discipline since adulthood (> 18 years), and the median training years for all masters athletes was 49.8 years. All participants provided written informed consent.

### Assessments

#### Anthropometry

Total body composition was assessed by dual-energy X-ray absorptiometry (DXA) (Lunar Prodigy Advance, version EnCore 10.50.086; GE Healthcare, Little Chalfont, UK) with arms and legs fully extended in the supine position. The cross-sectional area (CSA) of the quadriceps muscles was obtained using magnetic resonance imaging (MRI) at the muscle motor point, around mid-muscle belly. A T1-weighted turbo 3D sequence on a 0.25-T G-Scan (Esaote, Genoa, Italy) with participants lying supine was used. Continuous transversal images with a 6-mm slice were acquired and analysed by using Osirix imaging software (Osirix medical imaging, Osirix, Atlanta, GA, USA) through tracing around the quadriceps muscles following the contour of the aponeurosis. The highest CSA was recorded as peak quadriceps CSA (PQCSA) [54].

#### Physical Function

Participants were instructed to sit in a testing chair with hip and knee joints positioned at 90 degrees of flexion. An isometric force dynamometer (purpose-built calibrated strain gauge, RS Components Ltd, Corby, UK) was fastened securely 30 cm below the centre of the knee joint which participants were asked to elicit force against to perform an isometric knee extension. To minimise the movement of the upper trunk, a belt across the pelvis was fixed at the position of the anterior superior iliac spines. Prior to the assessment, a warm-up practice of submaximal contractions was required. Participants were then asked to perform a maximal effort, accompanied by verbal encouragement and visual feedback. The process was repeated three times with 60 s rest intervals between each; the best effort was regarded as the maximum voluntary isometric contraction (MVC).

Hand grip strength was measured using a handheld dynamometer (Jamar, Sammons Preston Inc., Bolingbrook, IL, USA). After adjusting the width of the dynamometer for each participant, participants were instructed to squeeze against the handle as hard as possible for approximately 3 s. This process was repeated twice for each hand with 30-s rest intervals between each. The maximum contraction force was recorded in kilograms to the nearest 0.1 kg.

A Leonardo Jump Mechanography Platform (Leonardo Software version 4.2: Novotiec Medical GmbH, Pforzheim, Germany) was used to assess lower limb power from a countermovement vertical jump [55]. Participants were instructed to flex the knee joint with feet approximately 30 cm apart (slightly narrower than shoulder width) and to jump as high and forcefully as possible with hands placed on the waist. Each participant repeated the jump sequence three times with approximately 30 s rest in between each; the highest value for relative jump power (W/kg) was recorded for further analysis.

A “Timed Up and Go (TUG)” test required participants to stand from a seated position, walk a distance of 3 m (10 feet), turn around a cone, return to the chair and sit down again as quickly as possible. Time started with the command “GO” and stopped when the participants returned to their original seated position.

#### Hormone quantification

Following an overnight fast, a 10 ml venous blood sample was collected from each participant at ~ 0900 h. Samples were immediately centrifuged at 3200 rpm, for 20 min at 4 °C, carefully aliquoted and frozen at − 80 °C for future analysis. The serum concentrations of dehydroepiandrosterone (DHEA), DHEA sulphate (DHEAS), total testosterone (T), dihydrotestosterone (DHT) and total estradiol (E2) were obtained and analysed using a liquid chromatography mass spectrometry high resolution system.

#### Intramuscular electromyography

**Fig. 1:**
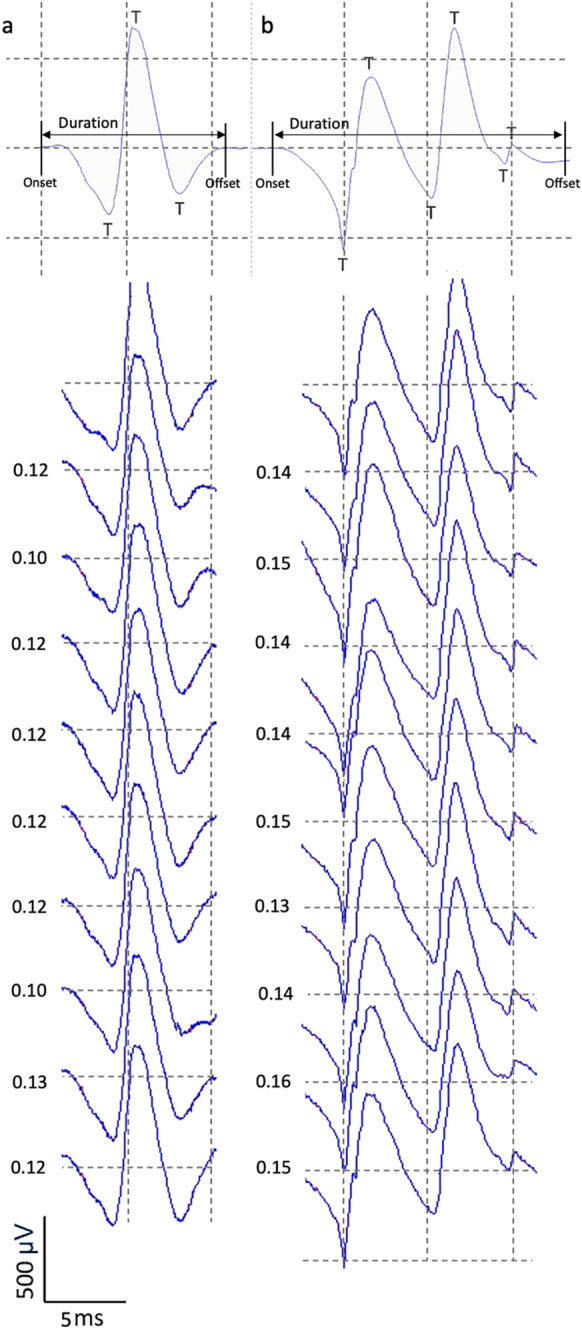
Example MUP templates (top) and 10 consecutive observations of the same MUP (bottom, raster plot) used to determine MUP duration, complexity (number of turns (T)) and firing rate. Inter-discharge intervals (IDIs, seconds) are shown to left of each MUP in the raster plot, corresponding to a firing rate of approximately 10.1 Hz (**a**) and 8.8 Hz (**b**)

The iEMG data were obtained through a disposable intramuscular concentric needle electrode (Model N53153; Teca, Hawthorne, NY, USA) inserted into VL at approximately 1–2 cm depth around the motor point. The signals were displayed and recorded in real-time via Spike2 software (Version 8.01), sampled at 25 kHz and bandpass filtered from 10 Hz to 10 kHz and stored for future offline analysis. The iEMG data were collected during a sustained voluntary isometric contraction lasting 12–15 s at 25% MVC with a target line displayed on a screen in front of the participants. Participants had ~ 30 s rest between each contraction. To avoid repeat sampling of the same MUs, after each contraction, the needle electrode was repositioned by rotating 180° and withdrawing by approximately 10–25 mm to obtain a minimum of 6 recordings from spatially distinct areas (from deep to superficial portions) [56]. The iEMG signals were analysed and converted into motor unit potential trains (MUPTs) using decomposition based quantitative electromyography software (DQEMG) [57]. Extracted MUPTs with fewer than 40 motor unit potentials (MUP) were excluded. We have previously reported MUP size parameters in a similar cohort as a direct group comparison [56]. Here, we report MU firing rate (FR), MUP duration and MUP complexity (number of turns) (Fig. 1) [16].

### Statistical analysis

**Fig. 2:**
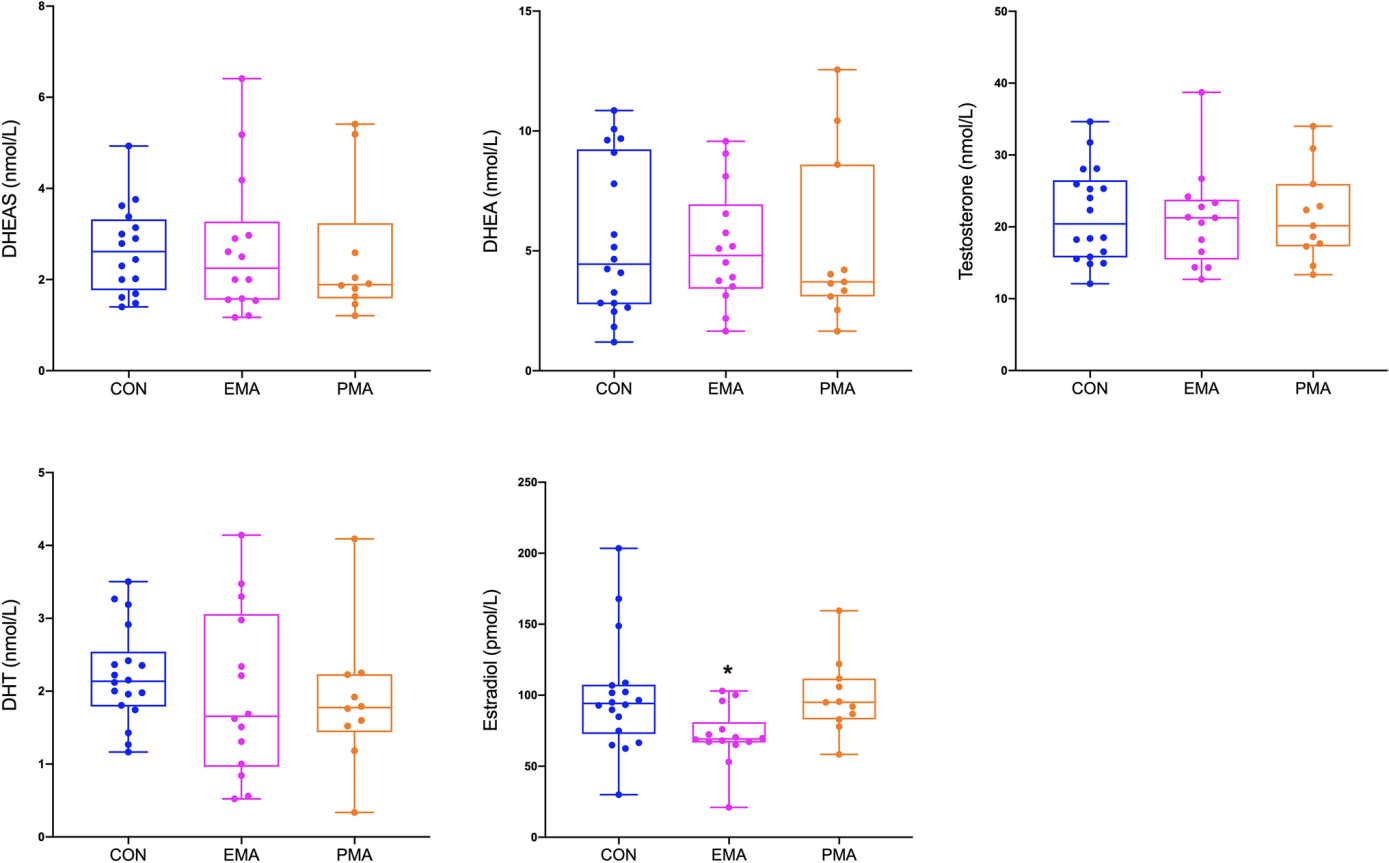
Circulating sex hormone levels among controls (CON), endurance masters athletes (EMA) and power masters athletes (PMA). ^*^p < 0.05. vs. CON and PMA

**Fig. 3:**
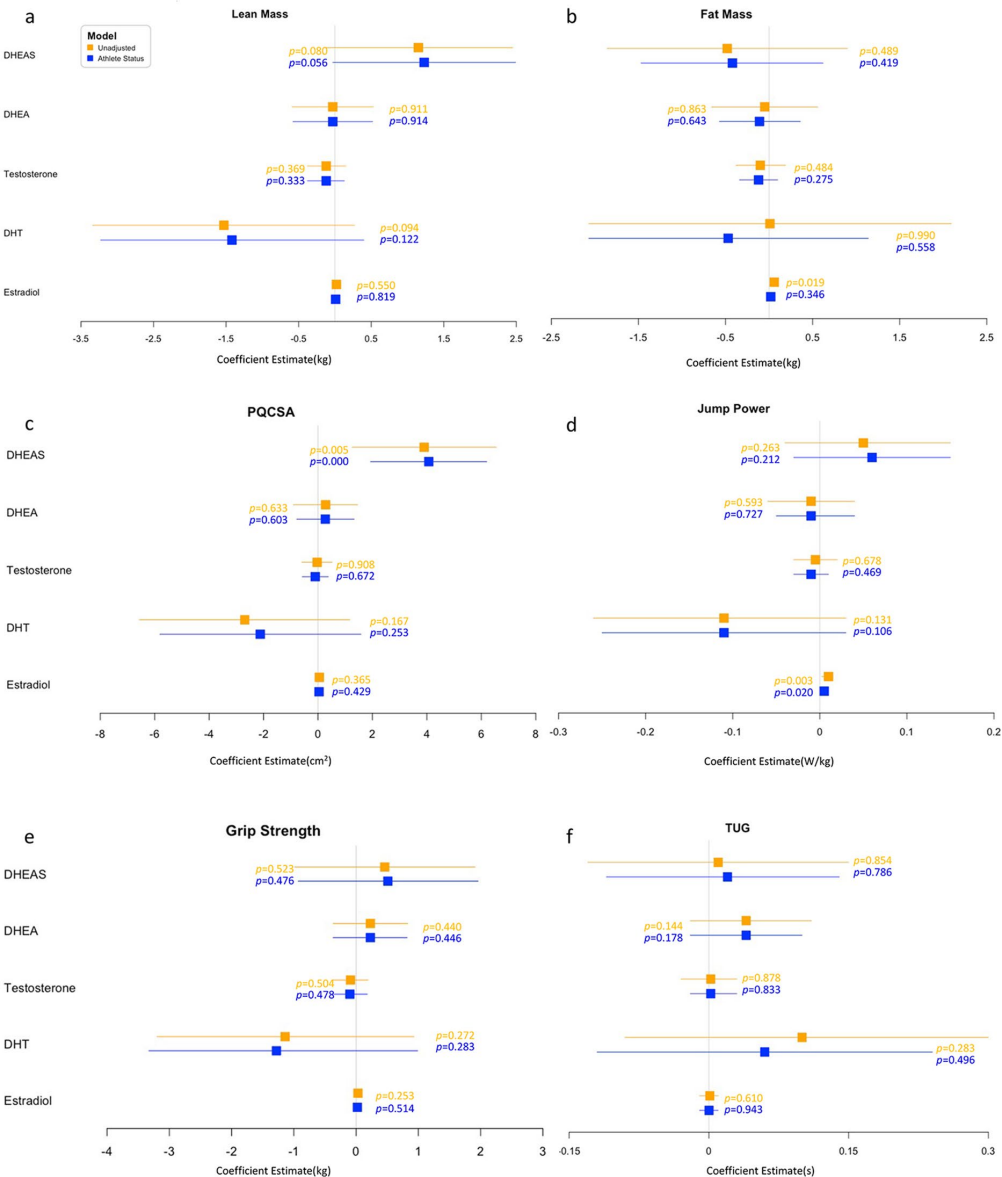
Forest plot for regression coefficient estimate (beta and 95% confidence interval) for unadjusted (orange) and adjusted (+ athletic status, blue) associations between hormone levels and physical function parameters in trained and untrained older adults. Beta represents the difference in outcome for 1-unit change in predictor (endocrine parameters). PQCSA, peak quadriceps cross-sectional area; TUG, timed up and go; DHEAS, dehydroepiandrosterone sulphate; DHEA, dehydroepiandrosterone; DHT, dihydrotestosterone. **a** Learn mass; **b** Fat mass; **c** PQSA; **d** Jump power; **e** Grip strength; **f** TUG

**Fig. 4:**
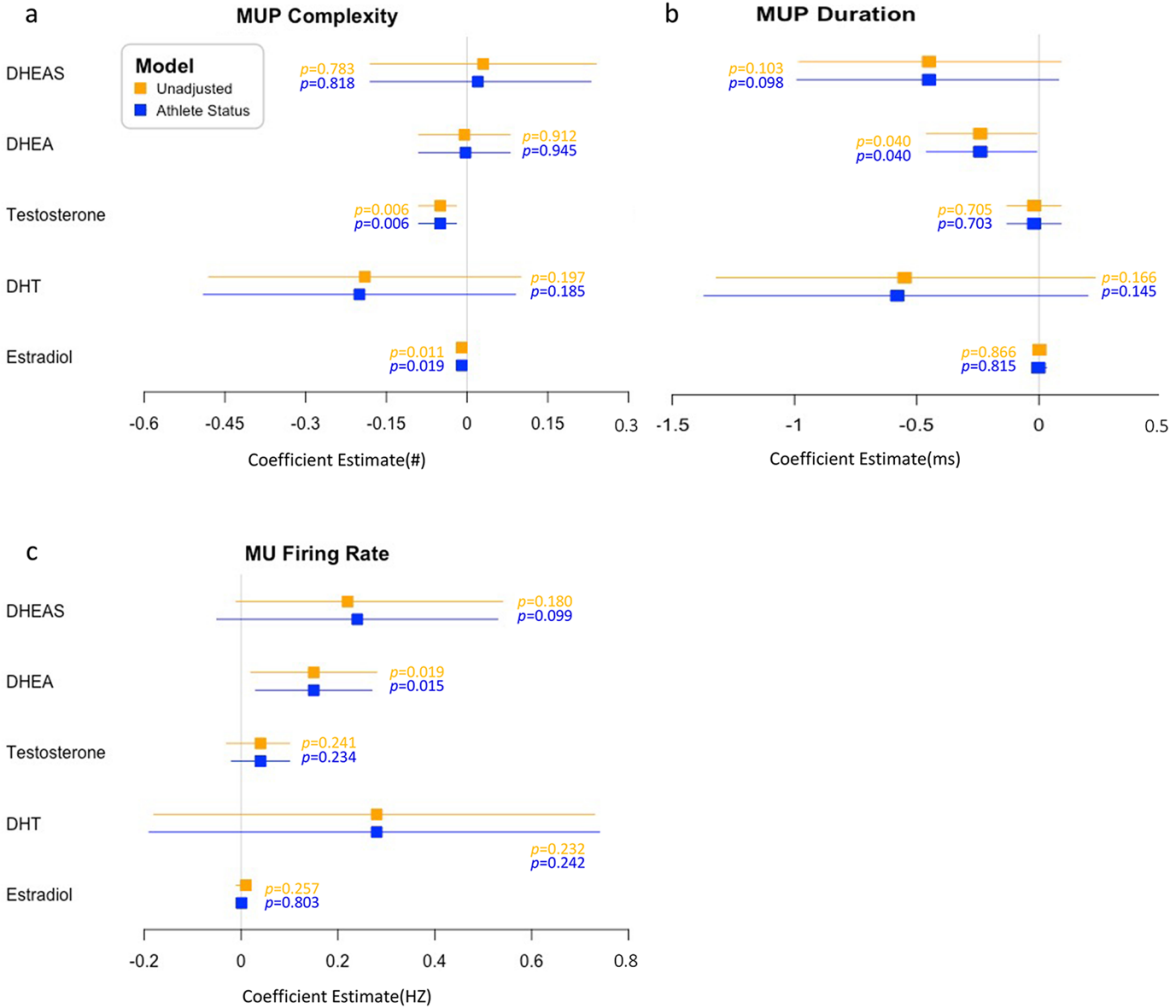
Forest plot for regression coefficient estimate (beta and 95% confidence interval) for unadjusted (orange) and adjusted (+ athletic status, blue) associations between hormone levels and motor unit (MU) features in trained and untrained older adults. Beta represents the difference in outcome for 1-unit change in predictor (endocrine parameters). MUP, motor unit potential; MUP complexity is defined as the number of turns; DHEAS, dehydroepiandrosterone sulphate; DHEA, dehydroepiandrosterone; DHT, dihydrotestosterone. **a** MUP complexity; **b** MUP duration; **c** MUP firing rate

Descriptive statistics in men by athletic status are presented as *mean* ± *standard deviation (SD)*. Between-group differences in functional measures and circulating hormones were assessed using *one-way ANOVA* followed by *Tukey’s *post hoc analysis. As multiple MUs were recorded from each participant, *multi-level linear regression models* were used to investigate the associations between hormones and MU properties, with each individual being regarded as an independent cluster and athletics status as a covariate. These results are displayed as coefficients estimate (beta and 95% confidence intervals) and *p* values. Significance was assumed when *p* < 0.05. All statistical analyses were performed using STATA-version 16 SE software (StataCorp, College Station, Texas) and the Figs. 2, 3, and 4 were created in RStudio version 4.0.2.

## Results

Forty-three men were included in the analyses, consisting of 18 elderly controls (mean ±* SD* age: 70.7 ± 3.7 years), 14 masters endurance athletes (68.6 ± 3.6), and 11 masters power athletes (70.5 ± 6.8) (Table 1).

**Table 1:** Participant characteristics by athletic status

	Control (CON)	Masters Endurance athletes (EMA)	Masters power athletes (PMA)
No	18	14	11
Age, y	70.7 ± 3.7	68.6 ± 3.6	70.5 ± 6.8
*Physical properties*			
Lean mass, kg	54.95 ± 5.3	54.48 ± 5.9	58.48 ± 4.0
Fat mass, kg	**16.87 ± 4.7**^**b**^	7.92 ± 3.3	**13.80 ± 5.3**^**b**^
PQCSA, cm^2^	62.2 ± 7.4	64.8 ± 11.0	**75.8 ± 12.4**^**ab**^
Grip strength, N	43.2 ± 6.3	41.0 ± 4.9	44.3 ± 5.2
Jump power, W/kg	2.47 ± 0.37	2.29 ± 0.31	**2.79 ± 0.56**^**b**^
TUG, s	5.91 ± 0.43	**5.41 ± 0.42**^**a**^	**5.36 ± 0.66**^**a**^
*MUP features*			
Complexity (no. of turns)	4.23 ± 0.81	4.51 ± 0.93	4.25 ± 1.1
Duration, ms	16.3 ± 1.87	16.15 ± 3.01	15.72 ± 2.07
Firing rate, Hz	**9.11 ± 1.19**^**b**^	8.30 ± 1.01	**9.61 ± 1.69**^**b**^

Data are mean ± standard deviation

*PQCSA*, peak quadriceps cross-sectional area; *TUG*, timed up and go; *MUP*, motor unit potential

The values in bold in the tables reflect statistically significant (*p* < .05) differences between groups. ^a^Significant difference to CON; ^b^significant difference to EMA. All MUP features were recorded at 25% MVC

Power masters athletes had greater muscle size than endurance and controls (both *p* < 0.05). There was no difference in lean mass or grip strength between groups. Power athletes exhibited greater jump power than endurance (*p* = 0.014), with no difference compared to controls (*p* = 0.134). Both endurance and power masters athletes had better TUG performance (*p* < 0.05) than their age-matched controls. Endurance athletes had lower fat mass and MU FR compared to controls and power athletes (*p* < 0.001). There were no significant differences in MUP duration or complexity between the groups (Table 1).

Lower levels of E2 were observed in endurance masters athletes when compared to controls (*p* = 0.016) and power athletes (*p* = 0.036). There were no differences in serum concentrations of DHEAS, DHEA, T, or DHT between the three groups (Fig. 2).

After adjusting for athletic status, for every unit increase in DHEAS, PQCSA increased by 4.07 cm^2^ (95% CI, 1.93 to 6.20, *p* < 0.001) (Fig. 3c). Similarly, for every unit increased in E2, jump power increased by 0.005 W/kg on average (95% CI, 0.001 to 0.01, *p* = 0.020) (Fig. 3d). Moreover, E2 was positively related to fat mass (*β* = 0.06; 95% CI, 0.01 to 0.11; *p* = 0.019) (Fig. 3b), becoming non-significant in adjusted models (*p* = 0.346). There were no significant relationships between any circulating sex hormones and lean mass (Fig. 3a), grip strength (Fig. 3e) or TUG (Fig. 3f) after adjustment for athletic status. (The detailed coefficient estimates can be found in Supplementary Material Table S1).

In both unadjusted and adjusted (for athletic status) analysis, DHEA showed a positive association with MU FR (*β* = 0.15; 95% CI, 0.02 to 0.27; *p* = 0.019) (Fig. 4c), and negative associations with MUP duration (*β* =  − 0.24; 95% CI, − 0.46 to − 0.01; *p* = 0.040) (Fig. 4b). Both T (*β* =  − 0.05; 95% CI, − 0.09 to − 0.002; *p* = 0.006) and E2 (*β* =  − 0.01; 95% CI, − 0.02 to − 0.002; *p* = 0.019) were negatively associated with MUP complexity (Fig. 4a). No significant relationships were observed between DHT and any MUP features (The detailed coefficient estimates can be found in Supplementary Material Table S2).

## Discussion

To our knowledge, this is the first study using combinations of MRI, DXA, intramuscular EMG and mass spectrometry techniques to explore associations between circulating sex hormone levels and MU characteristics in elite masters athletes. Although there was no difference in androgen concentrations across our groups, we show that power masters athletes generally had more favourable physical characteristics. We demonstrate that DHEA has a positive association with MU FR in elderly men. Additionally, the identification of an association between T levels and reduced MUP complexity suggests decreased electrophysiological temporal dispersion (increased activation synchronicity of MU fibres) in those with higher T levels. We also demonstrate that estrogen levels are positively associated with muscle power in both untrained and highly active older men.

Both longitudinal and cross-sectional studies have reported a downregulation of DHEA and its sulphate with ageing [24–26, 28], which has been suggested to be an independent predictor of muscle strength, muscle mass or muscle quality in elderly men and women [58, 59]. Chronic resistance exercise training has the benefit of elevating plasma and/or muscle levels of DHEA and T, and concurrently induces muscle size in older men [28, 32]. However, although a 12-week resistance exercise training regime appeared to attenuate age-related hormone reductions, there was no significant correlation between hormone levels and muscle strength or muscle mass [28]. Somewhat contrary to this, our study did show a positive association between DHEAS and quadriceps muscle size in old controls and elite athletes, which was independent of athletic specialism. Although observational, our findings further support previously reported associations between androgenic hormones and muscle size in older males [58].

In addition to its positive effects on cognition [60], notable evidence to date demonstrates that DHEA acts as a neurosteroid, regulating the motility and/or growth of neocortical neurons in the central nervous system [61]. DHEA is also known to influence neuronal excitability via the modulation of neurotransmitter receptors, such as N-methyl-D-aspartate (NMDA), gamma-aminobutyric acid type A (GABA_A_), and sigma receptors [62, 63]. Additionally, DHEA also contributes to neurogenesis and neuroprotection by mediating brain-derived neurotrophic factor (BDNF) [64, 65], which further regulates axonal regeneration, neuromuscular connections and ultimately, muscle force production. Increases in generation of force rely, in part, on MU FR, which responds differently to ageing and exercise training [66, 67]. Several studies have reported an apparent age-related decrease in MU FR, negatively influencing force production [11, 16, 68–70], and MU FR can be altered in response to exercise in young [71, 72] and older [73] people. The relationships between DHEA and MU FR with muscle strength have been established separately in humans [58, 73], and the positive associations between DHEA and MU FR during a submaximal contraction shown here highlight DHEA as a potential therapeutic intervention to increase MU FR, known to decrease with age and a probable factor in limiting neuromuscular function [11].

The number of MUP turns reflects the level of complexity of the MUP; greater turns indicate greater electrophysiological temporal dispersion. Notably, higher DHEA levels were associated with shorter MUP durations, also a measure of temporal dispersion. The negative associations between androgens and MUP temporal dispersion may be explained by greater MU fibre activation synchronicity or smaller conduction time differences along axonal branches and/or MU fibres, which is partly attributable to fibre conduction velocity [74–76]. Animal studies have demonstrated that androgens positively influence neural plasticity and axonal regeneration following nerve injury [77–79], and the potential ability of androgens to accelerate MU remodelling relies on the existence of androgen receptors (AR) [80], and androgen/AR signalling may improve neural transmission, motoneuron soma and dendrite size, and nerve regeneration [81]. Importantly, ARs are expressed in both motoneurons and muscle fibres, and may influence the release of synaptic vesicles and neurotransmitters at pre- and post-synaptic elements of NMJs directly or indirectly [48]. Androgen administration in animal models significantly expanded the pre- and post-synaptic elements of NMJs in fast twitch fibres, resulting in the improvement of neuromuscular transmission [47]. Although we did not directly quantify parameters related to androgen/AR signalling in these older males, long-term physically trained athletes exhibited similar levels of circulating androgens to untrained controls.

Circulating levels of E2 are primarily dependent on testosterone in males, converted via aromatisation partly occurring in adipose tissue. Although there were no differences in T concentrations, levels of E2 were lower in the endurance group when compared to controls and power athletes, which suggests an altered T:E2 ratio in the endurance athletes. Furthermore, the significant association between E2 and fat mass was not apparent when adjusting for athletic status, indicating the form of training influenced this relationship and this may be attributable to the lower fat mass in endurance athletes [82] and potentially, their lower levels of aromatase activity [83, 84].

Previous studies of older females reported a greater improvement in muscle strength and power in those receiving estrogen hormone therapy [85, 86], and here we report a similar association in older men. Moreover, these associations remained significant in follow-up analyses when adjusting for T, the precursor of E2, indicating total T concentrations do not influence this association. Mechanistic insight from animal models highlights marked improvements in myosin binding with estrogen hormone therapy [87], which may also extend to humans. For example, when estrogens were diminished, significant decrements in force generation were observed, and restored by hormone replacement [88]. Although predominantly associated with female neuromuscular health, E2 has several functions via both alpha and beta E2 receptors. Activation of both ERs promote a beneficial effect on bone health as well as playing an important role in regulating metabolic pathways and adipose tissue functions [89]. Moreover, ER-beta knockout mice models highlighted the importance of this receptor in the regulation of skeletal muscle growth and regeneration [90]. We have previously reported that both masters power and endurance athletes exhibited larger MUP size compared to age-matched controls, indicating a greater level of MU expansion [53], with no difference between endurance and power athletes, and that exercise has a range of established benefits on neuromuscular health. Other than E2, the current data shows long-term exercise training has minimal effects on circulating hormone levels in this age group. Taken together, these data suggest aspects of MU remodelling occurring in response to lifelong exercise do so independently of changes in circulating hormones.

### Strengths and limitations

This is the first study to investigate the relationship between hormone levels and MU characteristics in elite endurance and power masters athletes who were current competitors within their respective disciplines. As there were multiple MUs sampled during each muscle contraction, we used a multi-level mixed-effect linear regression model, allowing MU parameters to be clustered to an individual and overcome within-individual variability. However, the sample size is limited in this rare elite athlete cohort. It should be noted that only males were recruited into our study, and there is a lack of convincing evidence to explain the underlying mechanism of hormones on MU characteristics in females. Our study cannot provide evidence for causality between circulating sex hormone levels and neuromuscular characteristics.

## Conclusions

This study highlights the associations between circulating sex hormones and MU properties in older men. DHEA was positively associated with MU FR in these older men, a key component of muscle force generating capacity. Higher T levels were associated with reduced MUP complexity, indicating reduced electrophysiological temporal dispersion, which is related to reduced differences in conduction times along axonal branches and/or MU fibres. Although evident in males only, this work highlights the potential of hormone administration as a therapeutic interventional strategy specifically targeting the human neuromuscular system in older age.

## Supplementary Information

Below is the link to the electronic supplementary material.

Supplementary file1 (DOCX 25 KB)

## Data Availability

The data generated and analysed during the current study are available from the corresponding author upon reasonable request.
