# Supplementary material for: Circulating testosterone and dehydroepiandrosterone are associated with individual motor unit features in untrained and highly active older men
Source: GeroScience. 2021 Dec 3;44(3):1215–28. doi: 10.1007/s11357-021-00482-3 (PMC9213614; doi:10.1007/s11357-021-00482-3)
Supplement: Supplementary file 1 — Supplementary file1 (DOCX 25 KB) [file 11357_2021_482_MOESM1_ESM.docx]

**Supplementary Material**

| **Table S1. Unadjusted and adjusted regression relationships between hormone levels and physical properties** | | | | | | | | | | | | | | | | | | | | | | | | | | |
| --- | --- | --- | --- | --- | --- | --- | --- | --- | --- | --- | --- | --- | --- | --- | --- | --- | --- | --- | --- | --- | --- | --- | --- | --- | --- | --- |
|  |  | Lean Mass | | | | Fat Mass | | | | PQCSA | | | | Jump Power | | | | Grip Strength | | | | | TUG | | | |
| Endocrine Parameter | Models | *Beta* | *p* | *95%CI* | | *Beta* | *p* | *95%CI* | | *Beta* | *p* | *95%CI* | | *Beta* | *p* | *95%CI* | | *Beta* | *p* | *95%CI* | | *Beta* | | *p* | *95%CI* | |
| DHEAS  (nmol/L) | Unadjusted | 1.15 | 0.080 | -0.15 | 2.45 | -0.48 | 0.489 | -1.86 | 0.90 | 3.90 | **0.005** | 1.25 | 6.55 | 0.05 | 0.263 | -0.04 | 0.15 | 0.46 | 0.523 | -0.99 | 1.91 | 0.01 | | 0.854 | -0.13 | 0.15 |
|  | Athletic Status | 1.23 | 0.056 | -0.03 | 2.49 | -0.42 | 0.419 | -1.47 | 0.62 | 4.07 | **0.000** | 1.93 | 6.20 | 0.06 | 0.212 | -0.03 | 0.15 | 0.51 | 0.476 | -0.93 | 1.96 | 0.02 | | 0.786 | -0.11 | 0.14 |
| DHEA  (nmol/L) | Unadjusted | -0.03 | 0.911 | -0.59 | 0.53 | -0.05 | 0.863 | -0.66 | 0.56 | 0.28 | 0.633 | -0.90 | 1.46 | -0.01 | 0.593 | -0.06 | 0.04 | 0.23 | 0.440 | -0.37 | 0.83 | 0.04 | | 0.144 | -0.02 | 0.11 |
|  | Athletic Status | -0.03 | 0.914 | -0.58 | 0.52 | -0.11 | 0.643 | -0.57 | 0.36 | 0.27 | 0.603 | -0.78 | 1.33 | -0.01 | 0.727 | -0.05 | 0.04 | 0.23 | 0.446 | -0.37 | 0.82 | 0.04 | | 0.178 | -0.02 | 0.10 |
| Testosterone  (nmol/L) | Unadjusted | -0.12 | 0.369 | -0.38 | 0.15 | -0.10 | 0.484 | -0.38 | 0.19 | -0.03 | 0.908 | -0.59 | 0.52 | -0.005 | 0.678 | -0.03 | 0.02 | -0.09 | 0.504 | -0.38 | 0.19 | 0.002 | | 0.878 | -0.03 | 0.03 |
|  | Athletic Status | -0.12 | 0.333 | -0.38 | 0.13 | -0.12 | 0.275 | -0.34 | 0.10 | -0.10 | 0672 | -0.58 | 0.38 | -0.01 | 0.469 | -0.03 | 0.01 | -0.10 | 0.478 | -0.38 | 0.18 | 0.002 | | 0.833 | -0.02 | 0.03 |
| DHT  (nmol/L) | Unadjusted | -1.53 | 0.094 | -3.34 | 0.27 | 0.01 | 0.990 | -2.07 | 2.09 | -2.69 | 0.167 | -6.56 | 1.17 | -0.11 | 0.131 | -0.26 | 0.03 | -1.14 | 0.272 | -3.20 | 0.93 | 0.10 | | 0.283 | -0.09 | 0.30 |
|  | Athletic Status | -1.42 | 0.122 | -3.23 | 0.40 | -0.47 | 0.558 | -2.07 | 1.14 | -2.12 | 0.253 | -5.81 | 1.58 | -0.11 | 0.106 | -0.25 | 0.03 | -1.28 | 0.283 | -3.33 | 1.00 | 0.06 | | 0.496 | -0.12 | 0.24 |
| Estradiol  (pmol/L) | Unadjusted | 0.02 | 0.550 | -0.04 | 0.06 | 0.06 | **0.019** | 0.01 | 0.11 | 0.05 | 0.365 | -0.06 | 0.15 | 0.01 | **0.003** | 0.002 | 0.01 | 0.03 | 0.253 | -0.02 | 0.08 | 0.001 | | 0.610 | 0.00 | 0.01 |
|  | Athletic Status | 0.01 | 0.819 | -0.05 | 0.6 | 0.02 | 0.346 | -0.02 | 0.07 | 0.04 | 0.429 | -0.06 | 0.14 | 0.005 | **0.020** | 0.001 | 0.01 | 0.02 | 0.514 | -0.04 | 0.08 | -2e-3 | | 0.943 | -0.01 | 0.01 |

| **Table S2. Unadjusted and adjusted regression relationships between hormone levels and motor unit features** | | | | | | | | | | | | | | | | |
| --- | --- | --- | --- | --- | --- | --- | --- | --- | --- | --- | --- | --- | --- | --- | --- | --- |
|  | |  | | MUP Complexity | | | | MUP Duration | | | | MU Firing Rate | | | | |
| Endocrine Parameter | Models | | *Beta* | | *p* | *95% CI* | | *Beta* | *p* | *95% CI* | | *Beta* | *p* | *95% CI* | |  |
|  |  | |  | |  |  |  |  |  |  |  |  |  |  |  |  |
| DHEAS  (nmol/L) | Unadjusted | | 0.03 | | 0.783 | -0.18 | 0.24 | -0.45 | 0.103 | -0.98 | 0.09 | 0.22 | 0.180 | -0.10 | 0.54 |  |
|  | Athletic status | | 0.02 | | 0.818 | -0.18 | 0.23 | -0.45 | 0.098 | -0.99 | 0.08 | 0.24 | 0.099 | -0.05 | 0.53 |  |
| DHEA  (nmol/L) | Unadjusted | | -0.005 | | 0.912 | -0.09 | 0.08 | -0.24 | **0.040** | -0.46 | -0.01 | 0.15 | **0.019** | 0.02 | 0.28 |  |
|  | Athletic status | | -0.003 | | 0.945 | -0.09 | 0.08 | -0.24 | **0.040** | -0.46 | -0.01 | 0.15 | **0.015** | 0.03 | 0.27 |  |
| Testosterone  (nmol/L) | Unadjusted | | -0.05 | | **0.006** | -0.09 | -0.02 | -0.02 | 0.705 | -0.13 | 0.09 | 0.04 | 0.241 | -0.03 | 0.10 |  |
|  | Athletic status | | -0.05 | | **0.006** | -0.09 | -0.02 | -0.02 | 0.703 | -0.13 | 0.09 | 0.04 | 0.234 | -0.02 | 0.10 |  |
| DHT  (nmol/L) | Unadjusted | | -0.19 | | 0.197 | -0.48 | 0.10 | -0.55 | 0.166 | -1.32 | 0.23 | 0.28 | 0.232 | -0.18 | 0.73 |  |
|  | Athletic status | | -0.20 | | 0.185 | -0.49 | 0.09 | -0.58 | 0.145 | -1.37 | 0.20 | 0.28 | 0.242 | -0.19 | 0.74 |  |
| Estradiol  (pmol/L) | Unadjusted | | -0.01 | | **0.011** | -0.02 | -0.002 | 0.002 | 0.866 | -0.02 | 0.02 | 0.01 | 0.257 | -0.01 | 0.02 |  |
|  | Athletic status | | -0.01 | | **0.019** | -0.02 | -0.002 | 0.003 | 0.815 | -0.02 | 0.03 | 0.001 | 0.803 | -0.01 | 0.01 |  |
